# The association between work related factors and breastfeeding practices among Chinese working mothers: a mixed-method approach

**DOI:** 10.1186/s13006-019-0223-z

**Published:** 2019-06-27

**Authors:** Jiawen Chen, Tong Xin, Junjian Gaoshan, Qiuhong Li, Kaiyue Zou, Shihui Tan, Yuhan Cheng, Yuning Liu, Jingyi Chen, Hanyu Wang, Ying Mu, Li Jiang, Kun Tang

**Affiliations:** 1 School of Nursing, Peking University Health Science Center, 38 Xueyuan Road, Haidian District, Beijing, 100191 China; 2 Department of Global Health, School of Public Health, Peking University Health Science Center, 38 Xueyuan Road, Haidian District, Beijing, 100191 China; 3 United Nations Population Fund China office, 14 Liangmahe Nanlu, Chaoyang District, Beijing, 100600 China; 4 School of Basic Medical Sciences, Peking University Health Science Center, 38 Xueyuan Road, Haidian District, Beijing, 100191 China; 5 Department of Psychology, Northeastern University, 360 Huntington Ave, Boston, MA 02115 USA; 6 Department of Global Health and Population, Harvard University T. H Chan School of Public Health, 677 Huntington Ave, Boston, MA 02115 USA; 7 Institute for Medical Humanities, Peking University Health Science Center, 38 Xueyuan Road, Haidian District, Beijing, 100191 China; 8 Breast Surgery Department, Beijing Shijitan Hospital, Capital Medical University, Chaoyang District, Beijing, 100038 China; 9 Peking University People’s Hospital, No. 11 Xizhimen South Ave., Xicheng District, Beijing, 100044 China; 10 Research Center for Public Health, Tsinghua University, Haidian District, Beijing, 100084 China

**Keywords:** Work related factors, Workplace, Maternity leave, Maternal occupation, Occupation field, Employment status, Breastfeeding practices

## Abstract

**Background:**

Breastfeeding rates remain low in China and some mothers stop breastfeeding shortly after returning to work. Our study aimed to investigate the association between breastfeeding practices of working mothers and their employment status (formal versus informal) and occupational fields (agriculture related, industry related, and business and white collar). We also identified key work-related factors that influence breastfeeding practices in Chinese working mothers.

**Methods:**

This is a mixed-method research consisted of two components. We conducted a cross-sectional study of 10,408 breastfeeding mothers with children under 12 months old from 12 regions in China from July 2017 to January 2018. Multiple logistic regression was used to calculate adjusted odds ratios (AdjORs) and 95% confidence intervals (CIs) for breastfeeding practices. For the qualitative component, semi-structured interviews were conducted with 84 breastfeeding mothers in the study areas from July to December 2017, Content analysis was used for the qualitative component.

**Results:**

Agriculture related occupations were positively associated with early initiation of breastfeeding (AdjOR 1.32, 95% CI 1.15, 1.51), current breastfeeding (AdjOR 1.76, 95% CI 1.41, 2.20), ever breastfed (AdjOR 1.69, 95% CI 1.09, 2.62), exclusive breastfeeding (AdjOR 1.30, 95% CI: 1.04, 1.62), and predominant breastfeeding (AdjOR 1.72, 95% CI 1.44, 2.05). Business and white collar occupations were positively associated with early initiation (AdjOR1.38, 95% CI 1.23, 1.56) and ever breastfed (AdjOR 1.64, 95% CI 1.12, 2.39), and inversely associated with predominant breastfeeding (AdjOR 0.81, 95% CI 0.68, 0.95). For industry related and business and white collar occupations, informal employment was negatively related to current breastfeeding. In qualitative analysis, four main themes were developed to identify key work-related factors that influence breastfeeding practices: 1) employment benefits; 2) commute time; 3) workplace environment; 4) labor intensity. Mothers who experienced difficulties in one or more of the above would choose to lower breastfeeding frequency or stop breastfeeding.

**Conclusions:**

Having flexible work schedules and proximity of workplace to home can assist continuance of breastfeeding. Policies promoting supportive breastfeeding environment at work ought to be implemented. Additionally, informally employed mothers require more attention due to limited legal protection.

## Background

According to the Breastfeeding Enabling Environment Conceptual Framework, breastfeeding practice is primarily shaped on three levels, structural level, individual level and setting level [1]. Among all factors in the setting level, factors related to mothers’ occupations are one of the most important [2]. Numerous women worldwide spend a considerable amount of time on their jobs away from their children within the first year after giving birth [3]. A study conducted in the U.S. on working mothers suggested that the proportion of employed mothers who stopped breastfeeding early than recommended was higher than ever [4], showing only 25% of mothers with children under the age of one who had returned to work could continue breastfeeding their children for one month or longer. The percentage of working mothers who continued breastfeeding for six months, on the other hand, fell from 96% in 1983 to 31% in 2008 in Pakistan [5]. Returning to the workplace is one of the most common causes of mothers terminating breastfeeding all over the world [6].

Previous studies conducted in China showed that the overall breastfeeding rates in most provinces began to decline in the 1970s due to the widespread use of breast milk substitutes. Although there was some increment in the 1990s, the overall rates of breastfeeding still remained so low that most areas did not achieve the national target of “80 % exclusive breastfeeding rate” [7]. Furthermore, there is limited evidence on breastfeeding practices among working mothers in China. One of the studies conducted in Shanghai showed that most mothers returned to their workplace within 12 months after childbirth, and about 40% of them stopped breastfeeding after returning [8]. Another study conducted in Chengdu, a city in western China, indicated that upon returning to work, up to 74.5% of mothers continued breastfeeding their children until four to six months after birth. However, the percentage of mothers who continued for 6 to 12 months dropped to 35.2% [9].

Many work related factors such as fulltime maternal employment [10, 11], lack of paid maternity leave and lactation rooms, as well as inflexible work schedules [12] posed considerable challenges to breastfeeding practices for working mothers. Various interventions including enacting policies and action plans, raising awareness through media, and initiating social mobilization were proposed to promote breastfeeding practices [1]. Although most of the countries globally have policies protecting working mothers’ right to maternity leave, only 53% of these countries grant a recommended minimum duration of 14 weeks by International Labour Organization (ILO), and only 23% countries grant a duration of 18 weeks [13]. An analysis of national policies in 182 countries showed that paid breastfeeding breaks were guaranteed in 71% of the countries, unpaid breaks were offered in 4% of the countries, and 25% of the countries had no policy regarding maternity leave [14]. The *Special Provisions on Labor Protection of Female Employees* in China states that female employees have the right to enjoy 14 weeks of paid maternity leave as well as a one hour breastfeeding break at work each weekday. In China, a study in 2014 [15] suggested that the length of maternity leave, the intensity of occupation, and the establishment of lactation rooms at workplace were notably associated with total length and overall rate of breastfeeding. Studies showed that maternity leave, paid break guarantees, and the presence of lactation rooms increases breastfeeding rates [16, 17]. Given the potential differences in employment benefits and work conditions between a variety of occupation fields as well as between formal and informal employment, we believe breastfeeding practices of working mothers are affected by employment status and occupational fields. Yet, the relationships between employment status, occupational fields, and breastfeeding practices among the Chinese population are under investigated in the literature to the best of our knowledge.

Using data from a population-based survey of 10,408 breastfeeding mothers and subsequent qualitative interviews of a subgroup of these participants, this study aimed to investigate the association between breastfeeding practices of working mothers, their employment status (i.e. formal versus informal) and occupational field (i.e. agriculture related, industry related, and business and white collar). We also aimed to identify key work-related factors that influence breastfeeding practices in Chinese working mothers.

## Method

### Study design and participants

Using multistage sampling and probability proportional to size sampling, this population-based study was conducted in 12 randomly chosen county level regions, including four from urban cities, four from small and medium sized cities, two from rural areas, and two from poor rural areas in order to cover different levels of economic development and various geographic regions in China. In total, 10,408 mothers with children under 12 months of age were recruited in this study from July 2017 to January 2018.

For the qualitative part of the study using purposive sampling and snowball sampling techniques, mothers, their family members (husbands, mothers, and mothers-in-law) and stakeholders from 8 of the 12 regions, were recruited from July to December 2017. Based on the Breastfeeding Enabling Environment Conceptual Framework [1], such stakeholders included 10 healthcare professionals (physicians and nurses from maternal and child hospitals and primary care institutions), one private lactation consultant, and four infant formula sales agents. With the help from community health workers and village doctors, 84 mothers with children younger than 18 months were recruited in the study. Ethical approval was obtained from the Peking University Institutional Review Board. All participants provided informed consent. To encourage involvement, laundry detergent was given away as gift for each participant.

### Procedures

Each of the 12 regions established a quantitative data collection team consisted of five fulltime health workers with medical qualifications and field experiences. After registration and obtaining informed consent, the trained health workers administered a smartphone-based questionnaire to each participant. The questionnaire included questions regarding mothers’ sociodemographic and economic information, breastfeeding behavior and knowledge. The response rate was 90%.

After obtaining written informed consent, we conducted a semi-structured interview based on Breastfeeding Enabling Environment Conceptual Framework [1] and literature review results on social determinants, in the participants' homes or a quiet room in a community hospital where confidentiality was protected. For each interview, at least two interviewers with a background in public health and qualitative research were involved. Participants’ understanding on breastfeeding practices, influential factors, and their experiences were discussed during the interviews. All the interviews were recorded and transcribed for analysis.

### Quantitative data

#### Exposures

Occupational field and employment status were the two main exposures in the quantitative analysis. Out of 10,408 women recruited in this study, 10,384 (99.8%) mothers answered questions about their occupational status, and 659 were excluded for lack of detailed information. Hence, 9725 mothers were included for analysis.

Occupational field included: a) unemployed (i.e. freelancers, students, and housewives); b) agriculture-related (i.e. farmers, as well as jobs in forestry, animal husbandry, fishery, water conservancy, farm machinery operation, hunting, and etc.); c) industry related (i.e. miners, construction workers, drivers, and jobs involved in production, transportation, and etc.); d) business and white collar (i.e. business women, service workers, and jobs in service, sales, government offices or public institutions, and etc.)

Employment status was classified as formal (i.e. employment that offers various social security and benefits that include retirement insurance, medical insurance, unemployment insurance, workplace injury insurance, maternity insurance, and housing provident fund) and informal (i.e. employment without above-mentioned social security and benefits).

#### Outcomes

Four breastfeeding outcomes, including early initiation of breastfeeding (EIB), exclusive breastfeeding under six months (EBF), predominant breastfeeding under six months (PBF), and children ever breastfed (Ever BF), were collected based on the standardized questionnaire from the WHO [18]. The standardized questionnaire comprises of three modules: Household Roster, Initiation of breastfeeding (IBF) module and Infant and young child feeding (IYCF) module. Mothers were asked to recall the food they fed for their children in the last 24 h, and the final proportion was calculated among mothers with children 0–5 or 0–11 months old. EIB was defined as proportion of children born in the last 24 months who were put to the breasts within one hour of birth. EBF was defined as proportion of infants 0–5 months of age who were fed exclusively with breast milk. PBF was defined as proportion of infants 0–5 months of age who were predominantly breastfed, which mainly included exclusively breastfed children and who were breastfed by breast milk and water. Ever BF was defined as proportion of children born in the last 24 months who were ever breastfed. In addition, current breastfeeding (CBF) was defined as proportion of children born in the last 24 months who were then breastfed.

#### Other covariates

Mother and infant characteristics and socioeconomic status of the family were the two categories of covariates incorporated in the present study. Mother and infant characteristics included maternal age, pre-pregnancy BMI, parity, infant sex, and age. The information on gestational age, infant birthweight, delivery methods, and breastfeeding intention were also collected. Pre-pregnancy BMIs were calculated based on mothers’ self -reported weight and height before pregnancy. Mothers were asked how frequent was it that they were not willing to breastfeed. Answers options were as follows: a) never; b) rarely; c) sometimes; d) often; e) always. Breastfeeding intention was defined by the frequency of their willingness to breastfeed, and responses were grouped as never/rarely, sometimes/often, and always. Socioeconomic status of the family consisted of mothers’ level of education, region of residency (rural/urban), and status of residency (local/migrant).

### Data analysis

In quantitative analysis, descriptive statistics was used to report the background of participants by occupational field, mother and infant characteristics, family socioeconomic status, breastfeeding behaviors, and breastfeeding related workplace environment. The continuous variables were presented as means Standard Deviations (SDs) and the categorical variables as percentages to describe the participants.

The associations between occupational fields, employment status, and breastfeeding outcomes were analyzed using logistic regression models. Each outcome was modeled separately. All the adjusted odds ratios were presented with 95% confidence interval. Unemployment and formal occupation were chosen to be reference groups in all models. Adjustments were made for maternal education, maternal age, infant sex, parity, region, residency, and breastfeeding intention in the analysis of the associations between occupational fields and breastfeeding practices. In the analysis of the associations between employment status and breastfeeding practices, adjustments were made for maternal education, maternal occupation, maternal age, infant age, infant sex, parity, region, and breastfeeding intention. Models for analyzing the associations between occupational fields, employment status, and early initiation were plus adjusted for delivery method. Models for analyzing the associations between occupational fields, employment status, and current BF were plus adjusted for infant age. Mothers of agriculture related occupations were excluded in the analyses of associations between employment status and breastfeeding outcomes since 99% of agriculture related occupations were informal employment. For all the other tables, analyses were conducted in the whole population. All the analyses were conducted using SAS version 9.4 (SAS Institute, Cary, North Carolina, USA).

A deductive approach of content analysis was used to analyze qualitative data of 84 breastfeeding mothers. After transcription, research team members collaboratively coded interview transcripts with a list of predetermined themes based on the Breastfeeding Enabling Environment Conceptual Framework [1] and literature review results on social determinants of breastfeeding using the Dedoose qualitative research software version 8.0.36 (UCLA, Los Angeles, California, USA). New themes that repeatedly emerged from data were added to the coding list with consensus of the research team. The research team met regularly throughout the coding and analysis process to ensure inter-rater reliability. Each code application was compared for agreement between two members of the research team.

## Results

### Quantitative analysis

Table 1 shows the basic characteristics of the study population by occupational field. Out of 9725 women, 40.2% were unemployed, 19.9% worked in agriculture related fields, 2.4% worked in industry related fields, and 37.4% worked in business and white collar positions. The mean ages of those who were unemployed, in agriculture related fields, in industry related fields, and in business and white collar positions were 28.59 years (SD 4.98), 27.57 years (SD 5.51), 29.58 years (SD 4.02), and 30.53 years (SD 4.69), respectively. The distributions of pre-pregnancy BMI and infant sex were similar across all groups. Women who were working in business and white collar positions tended to be primiparous, have higher infant birthweight, live in urban areas, have higher maternal level of education, and hold formal employment. Women who were working in industry related fields and in agriculture related fields tended to be local residents. Women in agriculture related fields were more likely to have vaginal delivery as compared to industry related field workers. Over 90% of the mothers always had breastfeeding intention across all occupational fields.

**Table 1:** Characteristics of participants by each occupational field in survey study (*N* = 9725)

	Unemployed (*n* = 3910)	Agriculture related (*n* = 1937)	Industry related (*n* = 237)	Business and white collar (*n* = 3641)	Total (*n* = 9725)
Maternal age, year (SD)	28.59 (4.98)	27.57 (5.51)	29.58 (4.02)	30.53 (4.69)	29.15 (5.11)
Pre-pregnancy BMI, Kg/m2 (SD)	22.38 (9.07)	22.38 (5.13)	22.91 (4.38)	22.04 (6.91)	22.26 (7.37)
Gestational age, week (SD)	38.99 (1.43)	39.15 (1.37)	38.92 (1.26)	38.93 (1.45)	38.99 (1.41)
Infant birthweight, kg (SD)	3.42 (0.59)	3.27 (0.56)	3.34 (0.50)	3.42 (0.77)	3.36 (0.66)
Infant age, month (SD)	5.39 (3.44)	5.65 (3.41)	5.34 (3.37)	5.26 (3.42)	5.38 (3.44)
Infant sex, *n* (%)					
Male	2023 (51.74)	985 (50.85)	109 (45.99)	1808 (49.66)	4925 (50.64)
Female	1887 (48.26)	952 (49.15)	128 (54.01)	1833 (50.34)	4800 (49.36)
Parity, *n* (%)					
Primiparous	1624 (41.53)	729 (37.64)	71 (29.96)	1991 (54.68)	4415 (45.40)
Multiparous	2286 (58.47)	1208 (62.36)	166 (70.04)	1650 (45.32)	5310 (54.60)
Delivery method, *n* (%)					
Vaginal delivery	2220 (56.77)	1263 (65.19)	92 (38.94)	1994 (54.76)	5571 (57.29)
Cesarean section	1690 (43.23)	674 (34.81)	145 (61.06)	1647 (45.24)	4154 (42.71)
Region, *n* (%)					
Urban	2532 (64.76)	649 (33.51)	80 (33.76)	3116 (85.58)	6377 (65.57)
Rural	1378 (35.24)	1288 (66.49)	157 (66.24)	525 (14.42)	3348 (34.43)
Maternal education, *n* (%)					
Primary school and below	287 (7.34)	456 (23.54)	5 (2.11)	38 (1.04)	786 (8.08)
Middle School	1784 (45.63)	1261 (65.10)	122 (51.48)	386 (10.60)	3553 (36.53)
High/Vocational School	1009 (25.81)	159 (8.21)	59 (24.89)	527 (14.47)	1754 (18.04)
College and above	830 (21.23)	61 (3.15)	51 (21.52)	2690 (73.88)	3632 (37.35)
Resident status, *n* (%)					
Local	2314 (59.18)	1574 (81.26)	175 (73.84)	2024 (55.59)	6087 (62.59)
Migrant	1596 (40.82)	363 (18.74)	62 (26.16)	1617 (44.41)	3638 (37.41)
Employment status, *n* (%)					
Informal	/	1916 (98.92)	176 (74.26)	1089 (29.91)	6774 (69.66)
Formal	/	21 (1.08)	61 (25.74)	2552 (70.09)	2951 (30.34)
Had breastfeeding intention, *n* (%)					
Never/Rarely	29 (0.74)	17 (0.88)	1 (0.42)	29 (0.80)	76 (0.78)
Sometimes/Often	249 (6.37)	46 (2.38)	19 (8.02)	317 (8.73)	631 (6.50)
Always	3628 (92.88)	1870 (96.74)	217 (91.56)	3286 (90.47)	9001 (92.72)

Table 2 shows the numbers and percentages of mothers involved in various breastfeeding practices across different occupational fields. The distribution of percentages of mothers involved in different breastfeeding outcomes is highly variant across all occupational fields. Women who worked in business and white collar positions had the highest rates of early initiation and ever breastfed. Women who worked in agriculture related fields had the highest rates of current breastfeeding, exclusive breastfeeding, and predominant breastfeeding.

**Table 2:** The numbers and percentages of mothers involved in various breastfeeding practices across different occupational fields

	Unemployed		Agriculture related		Industry related		Business and white collar		Total	
	*n*	%	*n*	%	*n*	%	*n*	%	*n*	%
Early initiation	2672	68.34	1407	72.64	156	65.82	2755	75.67	6990	71.88
Current BF	3371	86.21	1780	91.89	216	91.14	3107	85.33	8474	87.14
Ever BF	3794	97.21	1894	97.88	230	97.05	3566	98.16	9484	97.69
Exclusive BF (0–6 months)	298	15.05	205	22.14	15	12.00	241	12.65	759	15.38
Predominant BF (0–6 months)	634	32.02	490	52.92	33	26.40	404	21.21	1561	31.62

Table 3 presents the associations between breastfeeding outcomes and mothers’ occupational field. Compared to the unemployed, those in agriculture related occupations were positively associated with EIB (AdjOR 1.32, 95% CI 1.15, 1.51), CBF (AdjOR 1.76, 95 CI 1.41, 2.20), ever BF (AdjOR 1.69, 95% CI 1.09, 2.62), EBF (AdjOR 1.30, 95% CI 1.04, 1.62), and PBF (AdjOR 1.72, 95% CI 1.44, 2.05). The business and white collar group was positively associated with early initiation (AdjOR 1.38, 95% CI 1.23, 1.56) and ever BF (AdjOR 1.64, 95% CI 1.12, 2.39) and inversely associated with PBF (AdjOR 0.81, 95% CI 0.68, 0.95). No significant association was found between breastfeeding outcomes and industry related occupations.

**Table 3:** Relationships between breastfeeding practices and occupational fields

	Adjusted Odds Ratios (95% Confidence Intervals)				
	Early initiation	Current BF	Ever BF	Exclusive BF (0–6 months)	Predominant BF (0–6 months)
Unemployed	1	1	1	1	1
Agriculture related	1.32 (1.15, 1.51)	1.76 (1.41, 2.20)	1.69 (1.09, 2.62)	1.30 (1.04, 1.62)	1.72 (1.44, 2.05)
Industry related	1.00 (0.75, 1.34)	1.19 (0.73, 1.94)	0.81 (0.35, 1.87)	0.77 (0.44, 1.34)	0.72 (0.47, 1.09)
Business and white collar	1.38 (1.23, 1.56)	0.98 (0.83, 1.15)	1.64 (1.12, 2.39)	0.95 (0.78, 1.17)	0.81 (0.68, 0.95)

*All models were adjusted for maternal education, maternal age, infant sex, parity, region (urban/rural), residency (local/migrant), and breastfeeding intention. Models for early initiation were plus adjusted for delivery method. Models for current BF were plus adjusted for infant age*

Table 4 shows the associations between breastfeeding outcomes and employment status (formal versus informal) in non-agriculture related occupations. Mothers with informal employment appeared to have lower odds of CBF (AdjOR 0.71, 95% CI 0.54, 0.94 for local mothers; AdjOR 0.69, 95% CI 0.51, 0.92 for migrant mothers) as compared to mothers who were formally employed. For migrant mothers recruited in the study, those with informal employment had significantly lower odds of EIB (AdjOR 0.59, 95% CI 0.38, 0.90), while other breastfeeding outcomes showed no significant associations with either formally or informally employed mothers.

**Table 4:** Relationships between breastfeeding practices and employment status (formal versus informal) in non-agriculture related workers, stratified by residency

		Adjusted Odds Ratios (95% Confidence Intervals)				
		Early initiation	Current BF	Ever BF	Exclusive BF (0–6 months)	Predominant BF (0–6 months)
Local	Formal	1	1	1	1	1
	Informal	0.93 (0.62, 1.39)	0.71 (0.54, 0.94)	0.87 (0.49, 1.55)	1.14 (0.78, 1.65)	1.33 (0.99, 1.78)
Migrant	Formal	1	1	1	1	1
	Informal	0.59 (0.38, 0.90)	0.69 (0.51, 0.92)	0.63 (0.35, 1.14)	1.29 (0.86, 1.92)	1.30 (0.94, 1.78)

*All models were adjusted for maternal education, maternal age, infant sex, parity, region (urban/rural), maternal occupation, and breastfeeding intention. Models for early initiation were plus adjusted for delivery method. Models for current BF were plus adjusted for infant age*

### Qualitative analysis

Table 5 shows the basic characteristics of the 84 interviewees with children younger than 18 months. Among them, 43% were unemployed, 4% were agriculture related workers, and 53% worked in business and white collar positions. Sixty four percent of the mothers were local residents and 36% were migrants.

**Table 5:** Characteristics of mothers in the interviews (*n* = 84)

Social demographic	Number (total = 84)	Percentage, %
Maternal occupations		
Unemployed	36	43
Agriculture-related	3	4
Business & White collar	45	53
Maternal education		
Primary school	5	6
Middle school	23	27
High school	17	20
College and above	39	47
Maternal region		
Local	53	64
Migrant	31	36
Household income		
≤ 50,000 yuan	23	27
50,000–100,000 yuan	29	35
100,000–200,000 yuan	20	24
≥ 200,000 yuan	12	14
Delivery method		
Vaginal delivery	41	49
Cesarean section	43	51
Infant sex		
Female	41	49
Male	43	51

Overall, analysis of the qualitative data revealed factors that influence mothers’ breastfeeding practices after they returned to work. Table 6 summaries the themes and sub-themes adopted from predetermined codes and merged during coding process. Four work related themes were identified: 1) employment benefits; 2) commute time; 3) workplace environment; 4) labor intensity.

**Table 6:** The influence of work-related factors on breastfeeding practices among working mothers

Theme	Sub-theme	
	Paid maternity leave	Breastfeeding breaks
Employment benefits	**+** paid maternity leaves for formally employed mothers	**+** legally required breastfeeding breaks for formally employed mothers
	- no guarantee for paid maternity leaves for informally employed mothers	- difficulty of utilizing breastfeeding breaks for mothers who work far from home
	- potential financial burden during maternity leave for informally employed mothers	
	Mother’s commute time	Proximity of family support
Commute time	**-** great distance from home and heavy traffic problems	**+** proximity and accessibility to family support for some informally self-employed workers
	**+** proximity of workplace to home	
	Space for lactation	Equipment for pumping breastmilk
Workplace environment	**-** lack of lactation rooms	**-** lack of basic equipment for pumping and storing breastmilk, such as electricity and refrigerator
	**+** some public space can be used for breastfeeding and pumping, but privacy and hygiene are not always ensured	
	Flexibility of work schedule	Stress from work
Labor intensity	+ flexible work schedule allows some working mothers to breastfeed during the day	- heavy and stressful workload
		- special work requirements that impeded breastfeeding attempts
	- special work requirements such as night shift	

*“+” means positive factors; “-” means negative factors*

#### Theme 1: Employment benefits

Formal employment can provide mothers with benefits ensured by law and regulations, including paid maternity leave and breastfeeding break time, while informal workers have to face various difficulties due to lack of employment benefits.

#### Sub-theme 1: Paid maternity leave

The *Special Provisions on Labor Protection of Female Employees* in China has specific requirements that female workers’ maternity leave should last for at least 98 days in total. Since maternity leave allows mothers to stay close to their children and breastfeed whenever needed, some mothers wished that maternity leave can be extended to six months as per WHO exclusive breastfeeding recommendation [19] or even longer. Additionally, mothers stated that it was tiring and difficult to continue breastfeeding after returning to work as they had to breastfeed babies at night and get up early for work.

*“I have been on maternity leave for six months now, but I would love to be on maternity leave for even longer, so that I can spend more time with the kids. Because I have night shifts from time to time, if I return to work, it means that I have to wean”. (QH10).*

*“It is so hard for us to continue breastfeeding*. *.*. *I am very tired and sleepy at work because I have to get up several times at night to feed her whenever she cries. I still have to get up early every morning to go to work*. *.*. *I wish I can have a longer maternity leave*. *.*.” *. (2JL19).*

2JL19 mother was formally employed with four month paid maternity leave. However, the maternity leave was not long enough for her to continue breastfeeding. For informally employed mothers, having maternity leave of desired length was even harder due to the unstable nature of their positions. Without legally protected maternity leave, these mothers’ work requirements, including employers’ personal opinion, social pressure and workload, limited their breastfeeding duration. Some of these self-employed mothers had to decrease breastfeeding frequency or wean early for returning to work due to financial burden.

*“Working at the supermarket was not exactly very convenient*. *.*. *I mean, the boss could understand that I need to breastfeed for the first several months after birth, but when it is time to wean you need to wean. It is common for people in this neighborhood to wean when the baby is 1 year old. This was also when I decided to wean. If the situation allows, you can continue breastfeeding. But when the situation does not work and you need to work, you need to wean”. (QH05).*

*“I knew the boss. He said I could take the break as long as I wished*. *.*. *But, uh, considering our future financial needs for the milk powder, and the tuition fees for schools, and so on, I told him I didn’t want go on leave for too long and I was willing to go to back to work soon*. *.*.” *. (GD04).*

#### Sub-theme 2: Breastfeeding breaks

The *Special Provisions on Labor Protection of Female Employees* in China also states that female workers who are feeding (including breastfeeding and artificial feeding) babies under one-year old should enjoy one-hour breastfeeding break at work each weekday. Breastfeeding breaks can greatly encourage mothers and their family members to overcome difficulties to continue breastfeeding. For example, some mothers we interviewed rented apartments near their workplace to continue breastfeeding during break time. Mothers who lived close to the workplace, especially those living in small cities and rural area, travelled back home during breastfeeding breaks. However, mothers who lived far from the workplace, especially those in big cities, found it hard to commute between workplace and home. Mothers who were informally employed may not have a breastfeeding break time at all because they were not under the protection of the above-mentioned regulations.

*“There is one hour in total for breastfeeding breaks, half an hour in the morning and half an hour in the afternoon*. *.*. *It is enough for me (to commute back and forth between workplace and home), because my home is very close to where I work*. *.*. *I drive home to breastfeed my baby every day because it only takes me two minutes”. (JL08).*

*“I don’t think of one hour breastfeeding break as particularly useful nowadays for working mothers, but it is better than nothing. I was thinking that the breastfeeding breaks might be more suitable for the past, just like the age of our mothers’ times. Many of them live very close to where they work back then, so they can go home at noon to feed the children and go back to work after feeding. But now, like me, I have to travel for about an hour from work to home, and so I am actually not able to feed my child during that only one-hour break time*. *.*. *But my colleagues and I can use that break to pump sometimes”. (BJ01).*

#### Theme 2: Commute time

Commute time was one of the factors that influenced breastfeeding practices for mothers who had resumed work. For those formally employed mothers, Commute time determined if breastfeeding was possible during breastfeeding breaks. For some informally self-employed workers, it determined if family support was accessible.

#### Sub-theme 1: Mother’s commute time

Mothers who lived close to their workplace could easily return home. The proximity of their home to workplace allowed the mothers to breastfeed their children in the middle of the day and therefore continue breastfeeding for longer duration. However, mothers who lived in urban settings, especially those living far from the workplace, often encountered traffic problems and found it difficult to breastfeed their children. Among those mothers who had longer commute time, the breastfeeding breaks appeared to be less essential and useful for them.

*“Just like a nurse in our department, she is given breastfeeding breaks to pump milk*. *.*. *She can also go home to breastfeed the baby because our city is very small*. *.*. *It only takes ten to twenty minutes to go back home and so she can come back in one hour. However, Beijing has a different conception of the distance. My brother lives in Beijing and I have been there several times. Life in Beijing is fast-paced and highly stressful*. *.*. *There are too many cars and there is always traffic jam. You cannot finish two things in one day”. (JL20).*

*“There is an hour of breastfeeding break for us, but it is a long commute between home and workplace. Going back and forth, it can add up to three to four hours. Going back at noon means not being able to go back to work for the afternoon. People in the unit would not let me just leave if there is so much work to do*. *.*.” *. (SD01).*

#### Sub-theme 2: Proximity of family support

Even though informal working mothers faced several barriers, such as lack of employment benefits, the unstable nature of the occupations may provide more possibilities and flexibilities. For some informally self-employed workers, without the limitation of a formal and rigid office space, support was more conveniently attained at their work environment. Their family members could use this opportunity to help out if they worked nearby. For example, a breakfast stand owner said the proximity of her workplace to home as well as the support from her family members provided her convenience to continue breastfeeding.

*“If the baby was hungry, his grandfather would take him to the breakfast stand so that I could breastfeed him*. *.*. *After breastfeeding, his grandfather would take him back home*. *.*. *The stand was just across the street. His grandfather could come to help me with the customers so that I could breastfeed the baby”. (GD11).*

#### Theme 3: Workplace environment

Compared to the rarely seen cases of breastfeeding children at work, carrying breast pumps to work was a more popular option for working mothers who hoped to continue breastfeeding. In this way, mothers may bring pumped milk home and ensure that neither their work nor their babies’ safe and nourishing food source was interrupted. This was also feasible for those mothers who could not commute back home during work. In addition, this arrangement raised concerns about whether the workplace provided space and equipment that can support the needs of breastfeeding mothers.

#### Sub-theme 1: Space for Lactation.

Social support and private and clean space were crucial to continue breastfeeding and pumping breast milk. 2 L19 mother collaborated with her husband during this process. In addition, JS03 mother managed to maintain good relationships with her colleagues who were supportive and willing to ensure privacy for her.

*“I pump the milk out and my husband drives to my office to pick it up every day*. *.*. *I am glad that our city is small so that I can continue breastfeeding. It would be much harder for me if I lived in Beijing”. (2JL19).*

*“At the beginning, I will pump the breast milk in the morning and in the afternoon. Then I will go home a little bit earlier than usual. My office is not as big as it used to be, but it is relatively private now. It used to be a complete open space. Now we can close the door. We only have four people in our department so I can pump in the office as long as I close the curtain*. *.*. *My colleagues and I are quite close, so my male colleagues would leave the room when I pumped. They were very nice”. (JS03).*

However, many mothers also expressed various concerns about breast pumping at workplace. Finding a safe and hygienic space while ensuring privacy was a common challenge. Many mothers stated that the lack of a lactation room at workplace was a significant factor in determining their breastfeeding practices. Although there were bathrooms or other vacant space that could be used for the same purpose, such as the staff rooms in hospitals and meeting rooms in banks, these environments were not clean and the vacant office rooms were deemed as less appropriate than lactation rooms. Lack of privacy and clean space made mothers nervous while nursing, which may adversely influence lactation [20].

*“It (a well facilitated lactation room) will at least provide me a private space where I can pump without rush. Now I can only pump in the bathroom, which is very dirty. What’s worse is that I have to rush because people are waiting outside*. *.*.” *. (BJ02).*

For mothers without better options, especially those informally employed and in unsatisfying environment, lack of private and hygienic space can be more challenging that encumbered breastfeeding practices.

*“In the supermarket where I worked, it is fine (to breastfeed) if there are just a few customers, but I always do it at home. It was inconvenient there, and it is very dirty. You have to wash your hands every time before breastfeeding, but you can hardly find a proper place to wash your hands there*. *.*. *There is only a small area separated from the outside by a curtain, where there is a bed for my boss and his families to take a break*. *.*. *I could use that space, I occasionally do*. *.*. *However, I seldom do that. I always breastfeed at home”. (QH05–1).*

#### Sub-theme 2: Equipment for pumping breastmilk

In addition to lack of private and clean public space, limited equipment at workplace was another challenge. Mothers often found it hard to have essential supplies workplace for breast milk storage, such as refrigerators and power outlets, and stated that these barriers had negatively influenced their lactation and led to early weaning.

“*Without a refrigerator, the storage and preservation of breast milk is also a big problem. Even if you have ice packs, the ice packs can easily melt. Breast milk must be kept at a low temperature”. (JS04–1).*

*“You have to go to the bathroom to pump, where there usually are no power outlets to charge the electric breast pump. Suction is thus less effective. It is easy to pump at home, but it is hard to pump it out in such settings*. *.*. *It is very stuffy in the bathroom*. *.*. *In addition, that place gets me uncomfortable*. *.*. *makes me feel bad*. *.*.” *. (JS04–2).*

#### Theme 4: Labor intensity

Most mothers noticed a close relationship between frequency of breastfeeding and lactation, which was well supported by previous research [21]. When mothers returned to work, their work schedules and workload may influence lactation by limiting on the frequency of breastfeeding or use of breast pumps. In addition, when some working mothers were under pressure, it became very tiresome and difficult for them to persist breastfeeding.

#### Sub-theme 1: Flexibility of work schedule

Mothers who had relatively flexible work schedules and control over their time, as exemplified by some mothers working as farmers, were able to breastfeed infants when they returned home during the middle of the day without affecting work. Additionally, some mothers in informal employment or worked in entry-level jobs might find it easier to ask for longer unpaid maternity leave without having to worry about losing their jobs or quit their jobs temporarily.

*“It takes me about five minutes to go to the socks factory by bus*. *.*. *My job is very flexible. The length of the leave is casual. You can ask for the leave as long as you want*. *.*. *I have been on leave for 15 months after I was pregnant*. *.*. *I knew the boss. Anyway, I can have a break at noon after I resume work. After feeding in the morning, I come back to feed at noon”. (JL07–1).*

*“It didn’t make much of a difference because I worked on the farm while the baby was asleep. It hardly delays the baby’s feeding*. *.*. *Baby is breastfed mainly. If I am too busy and away, my family will feed him formula milk and other supplementary food*. *.*. *We always put the baby at the first place*. *.*.” *. (GZ08).*

Flexibility with time was difficult for most working mothers. Some mothers who needed to work night shifts may decide to wean in advance or shortly after returning to work as lactation was negatively affected by irregular work schedules and less time with the baby.

*“Our colleagues all reported that the milk was less after work, because the frequency of breastfeeding practices decreased*. *.*. *I started to add complementary food before I resumed work*. *.*. *so the baby would not feel unaccustomed or hungry when I am not around*. *.*.” *. (JN07).*

#### Sub-theme 2: Stress from work

Many mothers knew the benefits and importance of breastfeeding, yet continue with breastfeeding became difficult and unrealistic after returning to work. Mothers suffered from heavy workload as their employers failed to delegate their responsibilities to others during their leave. This may negatively influence the optimal use of their paid maternity leave.

*“The production of breastmilk decreased as I started to work because there was much work waiting for me to finish. When I returned to work (from maternity leave), I worked overtime almost every day for about a month. Though I can get off work at 5:30pm, I usually had to stay for another one or two hours because there were a lot of work for me to finish. No one could help me*. *.*. *There were about six months’ work waiting for you. Your colleagues cannot replace you because the materials we make are very complicated*. *.*. *you can only finish it on your own”. (GD01).*

*“I could have over 100 days of paid maternity leave plus the university’s summer vacation. However, you know about working in a university*. *.*. *people started calling me one month after giving birth*. *.*. *was on and off*. *.*. *There was never a single minute that I am only carrying the responsibility of being a mother. No, there was not … balancing work, family and other stuff is difficult for a breastfeeding mom”. (JS06).*

In business and white collar occupations, the heavy workload after returning from maternity leave posed considerable amount of stress for working mothers. Some of them stopped breastfeeding after returning to work due to heavy workload and high level of stress, others who continued breastfeeding added supplements in food for the infants to meet nutritional needs. Many mothers said that the high level of stress had negative influence on breastmilk secretion.

*“My breastmilk was always enough (for my baby). However, my job is very tiring and lots of stuff in our company are continuously coming out. I think it (the stress) had some influences on production of breastmilk so the volume of my breast milk gradually decreased*. *.*. *After resuming work, I always try to satisfy his needs and accompany him*. *.*. *I still breastfeed him, but I have to add some milk powder for him now”. (JS06–1).*

Some occupational fields, including work at the counter or service window, imposed a different form of stress on working mothers. For example, some mothers stated that their work requirements made it hard for them to breastfeed or pump breastmilk at workplace because they were constantly engaged in communication with customers. Breastfeeding and pumping milk were regarded as inappropriate behaviors in these settings.

*“The inconvenience at workplace was one of the reasons. I worked at the first floor of the hall so I had to help people with lots of questions even though I had my own office room. It would be very inconvenient for me to suspend work from time to time and go inside to breastfeed him. I think it would be better for me to only breastfeed him at home”. (QH10).*

## Discussion

This study presented several important findings: firstly, in non-agriculture related occupations, negative associations were found between informal employment and current breastfeeding among both local and migrant populations. Secondly, in agriculture related occupations, 99% of which were informal, were positively associated with early initiation of breastfeeding, current breastfeeding, ever breastfed, EBF and predominant breastfeeding; and thirdly, business and white collar occupations were positively associated with early initiation and ever breastfeeding but was inversely associated with predominant breastfeeding.

In the current study, in non-agriculture related occupations, a negative association was found between informal employment and current breastfeeding among both local and migrant populations. According to our qualitative results, compared to informal employment, formal employment provided more employment benefits such as paid maternity leave and breastfeeding breaks. Additionally, informally employed mothers may have financial burden and may resume work shortly after childbirth out of this concern, giving rise to lower ever breastfeeding and predominant breastfeeding. A previous study showed that mothers were less likely to breastfeed for at least four months if they returned to work for financial reasons (adjusted Rate Ratio 0.86; 95% CI 0.80, 0.93) [11]. From qualitative interviews, results demonstrated that proximity of workplace to home and support from family members may be factors that encourage breastfeeding practices for some self-employed working mothers.

Compared with non-agriculture related occupations, we had different findings in mothers holding agriculture related occupations. Agriculture related occupations, 99% of which were informal, were positively associated with early initiation of breastfeeding, current breastfeeding, ever breastfeeding, EBF, and predominant breastfeeding. Additionally, the rate of early initiation of breastfeeding, current breastfeeding, ever breastfeeding, EBF, and predominant breastfeeding in agriculture related occupations were significantly higher than those in any other groups (*p* < 0.01). Flexible work schedule can be a main factor contributing to the breastfeeding practices seen in mothers holding agriculture related occupations. Additionally, agriculture related workers tended to live closer to their workplace. Although these mothers did not enjoy employment benefits, the flexible work schedule and proximity to home provided sufficient liberty and convenience to return home for breastfeeding. These results are consistent with those of previous studies that found part-time workers with more flexible work schedules are more likely to initiate breastfeeding [22] and to maintain breastfeeding practice for longer periods of time [23]. However, during certain farming seasons, mothers in agricultural occupations had to spend considerably more time working, consequently shortening the time breastfeeding and adding supplementary food to babies’ feeds. A qualitative study in Congo [24] also found that long working hours and heavy workload in agricultural fields could impede working mothers from feeding the infants optimally.

Many studies have found a significant association between mother’s returning to work and cessation of breastfeeding [25]. Many mothers stop breastfeeding earlier than recommended or intended because of several barriers after returning to work [26]. Lack of support at workplace is one of the most frequently cited barriers [11, 27]. In our study, we found that being in business and white collar occupations were positively associated with early initiation of breastfeeding and ever breastfeeding, but was inversely associated with predominant breastfeeding. Mothers holding business and white collar occupations had higher levels of education. Well educated mothers tended to seek out information about health practices from various sources [28, 29] and may be more conscientious about breastfeeding, which may lead to a higher ever breastfeeding rate. Yet, as demonstrated in the qualitative part of this study, heavy workload, high level of stress at workplace, and other barriers may negatively influence continuous breastfeeding. This may be especially true for mothers in business and white collar positions. Although some mothers might have breastfeeding breaks, distance from home or lack of lactation rooms at workplace hindered their current breastfeeding practices. Some studies have identified that having a designated lactation room (other than a bathroom, storage space, or equipment room) is a factor associated with breastfeeding success [30, 31]. Women need clean and private facilities where they can express breast milk at work [32]. Our findings suggest that lack of lactation rooms is one of the most serious barriers for working mothers to continue breastfeeding. Additionally, heavy traffic problems in urban metropolis is another barrier to breastfeeding, making it difficult for mothers to return home during breastfeeding breaks. Considering all the stated barriers, many mothers shared the concerns about the difficulties of expressing, storing, and transporting breast milk at work.

Our findings have practical implications and call for implementation of new policies to improve on building breastfeeding-friendly workplace. Having flexible work schedules and proximity of workplace to home seems to be helpful for the breastfeeding practices of mothers who return to their workplace. For those who cannot enjoy the flexibility and the proximity, an alternative is for the government to implement new policies promoting employers to provide clean and safe space for breastfeeding and pumping as well as build sufficient public lactation rooms equipped with a comfortable chair, a separate refrigerator, and power outlets for charging the electric breast pump. It is also important to protect job security and fair work responsibility of both formally or informally employed breastfeeding mothers, and that there are effective reporting systems available should concerns and issues arise. Intervention programs for working mothers dedicated at teaching effective stress management provided by healthcare professionals can also be beneficial.

### Strength and limitations

The present study is one of the limited number of studies focusing mainly on the association between breastfeeding practices of working mothers and their employment status and occupational fields in Chinese working mothers. This study combines quantitative and qualitative analyses and further investigates the interplay of different variables between employment status, occupations, and breastfeeding practices. The results of this study fill the knowledge gap in the associations between mothers’ occupational characteristics and their breastfeeding practices, providing evidence of the necessity of some related interventions and policies. Additionally, this study is based on large, nationally representative, population-based survey of 10,408 mothers. This large sample size provides for generalizability of the findings.

The present study has several limitations. Firstly, causal inference between maternal occupation, employment status and breastfeeding practices is limited due to the nature of the study being cross-sectional. A longitudinal study might provide more insights to establish causal relations. Secondly, as only three mothers in agriculture related occupations were interviewed in the qualitative part, our understanding of their opinions and experiences was limited. Thirdly, even though we strived to eliminate the bias by including all the confounders in the analyses, lack of information on some covariates such as smoking status limited our ability to perform more extensive analyses.

## Conclusions

In our present study, we explored the influence of such work-related factors as employment benefits, commute time, workplace environment, and labor intensity on breastfeeding practices. In order to improve the current situation in China, government should introduce policies to encourage employers providing breastfeeding-friendly work environment for working mothers. Additionally, the government should pay equal attention to the informally employed mothers, as there is a lack of regulations that offer legal protection for this large population.

## Data Availability

The dataset or transcripts are available from the corresponding author on reasonable request.

## Abbreviations

AdjORs: Adjusted Odds Ratios
CBF: Current Breastfeeding
EBF: Exclusive Breastfeeding
EIB: Early Initiation of Breastfeeding
Ever BF: Ever Breastfed
PBF: Predominant Breastfeeding
WHO: World Health Organization
